# African swine fever: A permanent threat to Indian pigs

**DOI:** 10.14202/vetworld.2020.2275-2285

**Published:** 2020-10-29

**Authors:** Sharanagouda S. Patil, Kuralayanapalya Puttahonnappa Suresh, Vikram Vashist, Awadhesh Prajapati, Bramhadev Pattnaik, Parimal Roy

**Affiliations:** 1 Indian Council of Agricultural Research-National Institute of Veterinary Epidemiology and Disease Informatics, Bengaluru, Karnataka, India; 2 Department of Animal Husbandry and Veterinary Services, Shimla, Himachal Pradesh, India; 3 One Health Center for Surveillance and Disease Dynamics, AIPH University, Bhubaneswar, Odisha, India

**Keywords:** African swine fever, Arunachal Pradesh, Assam, first outbreak, India, North eastern states, pigs, social and economic factors

## Abstract

India has 9 million pigs, of which 45% are in the North eastern (NE) states of India. Viral diseases affecting pigs are a major concern of mortality causing huge loss to the pig farmers. One such disease is African swine fever (ASF) that has already knocked the porous borders of NE states of India. ASF is a highly contagious devastating disease of pigs and wild boars causing 100% mortality. The causative agent African swine fever virus (ASFV) belongs to the genus *Asfivirus*, family *Asfarviridae*. Pig is the only species affected by this virus. Soft ticks (Ornithodoros genus) are shown to be reservoir and transmission vectors of ASFV. Transmission is very rapid and quickly engulfs the entire pig population. It is very difficult to differentiate classical swine fever from ASF since clinical symptoms overlap. Infected and in contact pigs should be culled immediately and buried deep, and sheds and premises be disinfected to control the disease. There is no vaccine available commercially. Since its first report in Kenya in 1921, the disease has been reported from the countries in Europe, Russian federation, China, and Myanmar. The disease is a threat to Indian pigs. OIE published the first report of ASF in India on May 21, 2020, wherein, a total of 3701 pigs died from 11 outbreaks (Morbidity - 38.45% and mortality - 33.89%) in Assam and Arunachal Pradesh states of India. ASF is non-zoonotic.

## Introduction

African swine fever (ASF) is a highly contagious, devastating disease of pigs and wild boars causing severe mortality. ASF is caused by African swine fever virus (ASFV), a genetically complex virus belonging to the genus *Asfivirus* of family *Asfarviridae* [1]. OIE has listed ASF as a notifiable disease. The disease was first described in the early 1900’s when the European pig breeds were introduced in Kenya colony [2] and later on disease entered in Europe (Portugal) in 1957 which was controlled quickly, but re-entered Portugal in 1960 and spread to Iberian Peninsula and rest of the Europe [3-7]. Further, progressing to Russian Federation in 2007 and spread to China in 2018, then to Vietnam and Myanmar and to India (Table-1) [8,9]. ASFV is spreading transcontinental and has apprehension of becoming a global pig health problem.

**Table 1:** Reports of ASF in Europe, Asia, and other countries since 1957 [8,9].

S. No	Name of Country	Year of Reporting
1.	Portugal	1957 and 1960
2.	Italy	1967
3.	Spain	1969
4.	Cuba	1971 and 1980
5.	France	1977
6.	Malta, Brazil, the Dominican Republic	1978
7.	Haiti	1979
8.	Belgium	1985
9.	Netherlands	1986
10.	Georgia, Armenia, and Russian Federation (RF)	2007
11.	Azerbaijan, Iran	2008
12.	Ukraine	2012
13.	Belarus	2013
14.	Lithuania, Poland, Latvia, and Estonia	2014
15.	Moldova, Irkutsk (RF), Czech Republic, Romania	2017
16.	China	August, 2018
17.	Mongolia	January, 2019
18.	Vietnam	February, 2019
19.	Cambodia	March, 2019
20.	Hong Kong	May, 2019
21.	North Korea	May, 2019
22.	Laos	June, 2019
23.	Philippines	July,2019
24.	Myanmar	August, 2019
25.	Indonesia	September, 2019
26.	South Korea	September, 2019
27.	Timor-Leste	September, 2019
28.	Papua New Guinea	March, 2020
29.	India	May, 2020

India has 9 millions of pigs (Table-2) [10] and North eastern (NE) states of India are having more than 45% of Indian pig population (Table-3) [10] mostly reared by economically disadvantaged people and pork is their staple food. Further, many NE states have porous borders sharing with Tibet, China, Myanmar, and Bangladesh. ASF has already caused a catastrophic effect in pigs in the neighboring countries of India that has cautioned policymakers to be alert in preventing the entry of disease. To safeguard the pig population and to have food security, keeping vigilance on the entry of ASFV in to our territory is of prime importance. A total of 160 new outbreaks were notified from August 21 to September 3, 2020, and that of total ongoing ASF outbreaks worldwide is 7,191 (including 3733 outbreaks in Romania and 1474 outbreaks in Vietnam) [8].

**Table 2:** Details of susceptible pig population in states other than NE states of India [10].

S. No	State	Pig Population
1.	Andaman and Nicobar	40,488
2.	Andhra Pradesh	91,958
3.	Bihar	3,43,434
4.	Chhattisgarh	5,26,901
5.	Goa	35,480
6.	Gujarat	658
7.	Haryana	1,08,240
8.	Himachal Pradesh	2,477
9.	Jammu and Kashmir	1,215
10.	Jharkhand	12,76,973
11.	Karnataka	3,23,836
12.	Kerala	1,03,863
13.	Madhya Pradesh	1,64,616
14.	Maharashtra	1,61,000
15.	Odisha	1,35,162
16.	Puducherry	880
17.	Punjab	52,961
18.	Rajasthan	1,54,808
19.	Tamil Nadu	66,772
20.	Telangana	1,77,992
21.	Tripura	2,06,035
22.	Uttarakhand	17,659
23.	Uttar Pradesh	4,08,678
24.	West Bengal	5,40,356

**Table 3:** Details of pig population at immediate risk of disease in NE states of India [10].

S. No	States	Pig population
1.	Arunachal Pradesh	271,463
2.	Assam	2,099,000
3.	Manipur	235,255
4.	Meghalaya	706,364
5.	Mizoram	292,465
6.	Nagaland	404,695
7.	Sikkim	27,320
8.	Tripura	206,035
	Total	4,242,597

OIE reported on May 21, 2020, a total of 11 outbreaks in Assam and Arunachal Pradesh states of India, wherein 3701 pigs were died due to ASF (Table-4) [11]. Keeping in view of the report on outbreak of ASF in India, this review discusses on the ASF, its epidemiology and transmission, pathogenesis, prevention, and control.

**Table 4:** Details of first report of African Swine Fever in India [11].

State	Place	Susceptible	Cases	Deaths
Assam	Pipolguri, Sissiborgaon, Dhemaji, Assam	2450	1897	1803
	Kotiyori, Demow, Sivasagar, Assam	289	163	117
	Khelowa, Sivasagar, Sivasagar, Assam	731	326	298
	Nitai-pukhuri, Demow, Sivasagar, Assam	642	62	45
	Bor-Tamuli-II, Pub-Chaiduar, Biswanath, Assam	689	317	283
	Gorchuk, Ward No.7, Kamrup Metro, Assam	153	22	22
	Bormukoli, Dergaon, Jorhat, Assam	2580	956	853
	Total	7534	3743	3421
Arunachal Pradesh	Huchang, Damsite, Naharlagun, Papum Pare, Arunachal Pradesh	419	156	103
	Bamin, Bilat, East Siang, Arunachal Pradesh	653	123	67
	Pasighat Town, Pasighat, East Siang, Arunachal Pradesh	1551	165	101
	D-Sector, Nirjuli, Doimukh, Papum Pare, Arunachal Pradesh	763	12	9
	Total	3386	456	280

## Etiology and Genome Organization

The causative agent of ASF is ASFV belonging to the genus *Asfivirus* of the family *Asfarviridae*. ASFV is the only virus listed in that genus [1].

**Figure-1:**
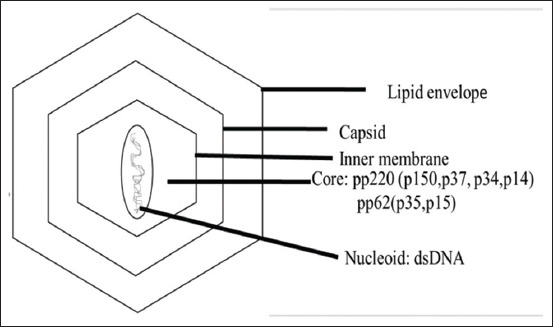
Structure of African swine fever virus [12].

**Figure-2:**
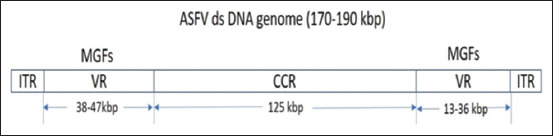
African swine fever virus genome organization: ITR: Internal terminal repeats, VR: Variable region, MGFs: Multigene families, CCR: Central conserved region [12].

ASFV is an enveloped virus (Figure-1) [12] having linear genome of double-stranded DNA of length between 170 kb and 190 kb in size containing 150-167 ORFs with a conserved central region of about 125 kb and variable ends (Figure-2) [12]. Five multigene families encoded by these variable regions contribute to the variability of the virus genome. The ASFV consists a central DNA with a thick protein layer called the core shell, an inner lipoprotein envelope surrounding the core and the icosahedral capsid (p72). The virions on maturity possess a lipid envelope acquired by budding from the plasma membrane. ASFV displays icosahedral symmetry with a diameter of 200 nm. During replication inside host cells, 60 structural proteins related to ASFV or more have been detected [13]. In infected porcine macrophages, 100 viral proteins or more have been identified, of which more than 50 of them react with sera from ASFV recovered pigs [14]. Different genotypes of ASFV have been grouped and recovered from variable severity of disease and there is only one serotype of ASFV detectable by antibody test. There are 24 distinct genotypes classified based on p72 protein.

Tandem repeat sequences within the B602L gene [15] and the intergenic region between the I73R and I329L genes [16] are sequenced to distinguish subgroups among closely related ASFV. Other genomic regions, namely, E183L (p54), CP204L (p30), and EP402R (CD2v) have been useful spatial epidemiological studies.

## Epidemiology

The spread and transmission of ASF are unique to each continent of Africa, Europe, and Asia. There are three cycles of ASF epidemiology such as sylvatic, tick–pig, and domestic, with addition of more factors, that is, soft *Ornithodoros* spp. ticks, wild African pigs (mainly warthogs), domestic pigs, and pig-derived products such as pork [17]. In the sylvatic cycle, virus is maintained between warthogs and soft ticks, without causing disease in the warthogs [18]. This may be the reason that the virus spilled over to domestic pigs causing disease. In the tick–pig cycle, ticks act as biological reservoir to maintain the virus and infectious agents gets transmitted among domestic pigs [19]. Such type of cycles was seen in parts of sub-Saharan Africa and also during the epidemic on the Iberian Peninsula in the ‘60s and ‘70s of the past century [20]. In the domestic cycle, natural reservoirs do not play a role but disease is transmitted in domestic herds from domestic pigs or pig products [21]. The new and fourth cycle of transmission named wild boar-habitat cycle (proposed) reported in some parts of Europe involves Eurasian wild boar, its habitat and their carcasses [22]. This fourth cycle is involving both direct transmissions between wild boar, and indirect transmission through the habitat [23]. Cold and moist climate favors persistency and human movement are the cause of transmission in domestic pigs [24]. Social and economic factors of pig husbandry are also associated with ASF outbreaks in the domestic cycle [25]. It may be hypothesized that the fourth cycle of ASF virus transmission may be attributed to the first outbreak in Assam and Arunachal Pradesh and subsequently domestic cycle might have played in disease transmission between domestic pigs through contaminated pig products/fomites.

### Wild boar-habitat cycle

The wild boar-habitat cycle is having both direct transmission between infected and susceptible wild boar and indirect transmission through carcasses in the area where wild boar habitat. Such areas get contaminated from infected wild boar carcasses, subsequent scavenging by other wild/other pigs [23,26], offer possibilities for both low-dose and high-dose infections, depending on carcass decomposition. It is also observed that the virus may be present in the carcass even if it is a single dead pig for long time and continue to get transmitted to the susceptible animals [27,28]. The interface contact between wild boar habitat and domestic pigs is critical in transmission of virus which might have caused first outbreak of ASF in India (hypothetical). Environmental persistence of the virus is favored by a cold and moist climate [22,23,26-28].

### Social and economic factors of pig husbandry associated with the domestic cycle

On confirmation of ASF in Assam and Arunachal Pradesh, culling of all susceptible pigs in-1 km radius of epicenter was started. Farmers lost thousands of pigs in this process in addition to those pigs died of disease. As there is no medicine or vaccine, disease got spread rapidly and farmers continue to lose their pigs. Pig-farmers, especially those rearing backyard pigs, had to reduce their holdings and even closing their units of late. Hence, pig husbandry in both states got in disarray and many farmers lost their job and there was no immediate financial support or relief from governments. Consumptions of pork also reduced. Sale of even healthy pigs reduced due to apprehension of the consumers that they may get the disease contracted otherwise it is not [29].

## Geographical Distribution of ASF

**Figure-3:**
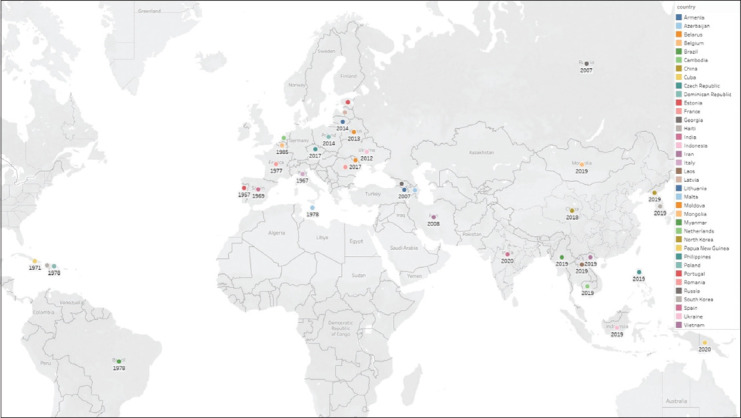
Countries that have reported African swine fever in Europe, Asia and other continents since 1957 [8,9].

**Figure-4:**
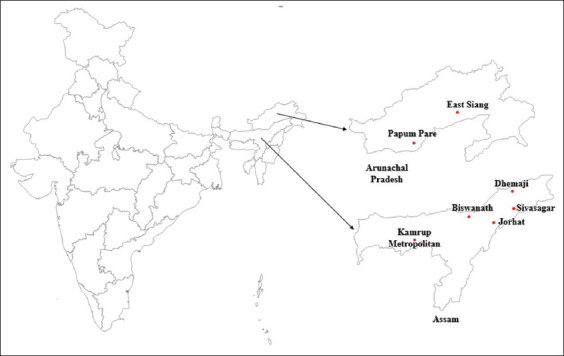
Locations of first reports of African swine fever in Arunachal Pradesh and Assam in India [11].

World Animal Health Information System of OIE maintains the outbreaks reports of ASF in different countries. Since 2016, a pattern of significant increase in the amount of outbreaks has been identified. ASF is present in the African, European, and most recently, the Asian continent (Table-5) [30]. Most recently there was an outbreak in Papua New Guinea (Oceania) during March, 2020 [8]. It was eradicated in the Americas in the 90’s [31]. Since 2016, 30% of the reporting countries and territories (60/201) have identified the disease as present (Table-6) [31]. The disease occurred for the first time in Europe, in Moldova in September 2016, then in June 2017 in Czech Republic, followed by Romania in July 2017 and later on in Hungary, and Bulgaria, in April and August 2018, respectively. A recurrence of the disease in wild boars was reported in Belgium in September 2018 (last event occurred and was resolved in 1985). In Asia, the disease was reported for the first time in China (People’s Republic of) in August 2018, Mongolia in January 2019, and then Vietnam in February 2019, Cambodia in March 2019, and Hong Kong (SAR-PRC) in May 2019, followed by Laos in June 2019, Myanmar in August 2019, Philippines in July, 2019, Korea (Rep. of) in September 2019, Timor-Leste in September 2019, Indonesia in November 2019, and more recently, Papua New Guinea in March, 2020 (Figure-3), and India in May 2020 (Figure-4) [30].

**Table 5:** Cases and losses due to ASF during 2016-2020 in three affected continents [30].

Region	Swine			

	Outbreak	Susceptible	Cases	Losses (dead and Culled)
Africa	128	2,13,795	61,459	85,539
Europe	4,271	18,59,480	6,25,269	1,383,372
Asia	9,928	81,07,951	1,15,309	6,733,791
Total	14,327	1,01,81,226	8,02,037	8,202,702

**Table 6:** List of the countries that reported ASF globally [31].

S. No	Name of the Country	S. No	Name of the Country
1	Angola	31	Latvia
2	Belgium	32	Lithuania
3	Benin	33	Madagascar
4	Bulgaria	34	Malawi
5	Burkina Faso	35	Mali
6	Burundi	36	Moldova
7	Cabo Verde	37	Mongolia
8	Cambodia	38	Mozambique
9	Cameroon	39	Myanmar
10	Central African Republic	40	Namibia
11	Chad	41	Nigeria
12	China (People’s Republic of)	42	Poland
13	Congo (Dem. Rep. of)	43	Papua New Guinea
14	Congo (Rep. of)	44	Philippines
15	Cote D’Ivoire	45	Romania
16	Czech Republic	46	Russia
17	Estonia	47	Rwanda
18	Gambia	48	Senegal
19	Ghana	49	Serbia
20	Guinea-Bissau	50	Slovakia
21	Greece	51	Sierra Leone
22	Hong Kong (SAR-PRC),	52	South Africa
23	Hungary	53	Tanzania
24	Indonesia	54	Timor-Leste
25	India	55	Togo
26	Italy	56	Uganda
27	Lao-PDR	57	Ukraine
28	Kenya	58	Vietnam
29	Korea (Dem. People’s Rep. of)	59	Zambia
30	Korea (Rep. of)	60	Zimbabwe

ASF in one wild boar is considered as a single outbreak which needs a targeted surveillance to be resolved immediately that is practiced in Europe. Africa and Asia have notified outbreaks in domestic pigs mainly, and few cases in wild boar (300 cases reported in Asia since August 2018). During this period, Europe accounted for the majority of outbreaks with 96% (9756) of all outbreaks, but the highest impact in terms of animal losses was reported in Asia (1711677 animals lost, which is 68% of the total global reported losses in this period) [30].

The contagiosity, tenacity, and case fatality rate due to ASFV in the population favors its persistence and transmission that becomes hurdle in its eradication programs [32]. Therefore, strict biosecurity measures need to be put in place as per OIE guidelines.

On May 21, 2020, OIE published the first report of ASF in two states of India, namely, Assam and Arunachal Pradesh. There were 4199 cases of ASF in a susceptible pig population of 1092 across five districts and two districts in Assam and Arunachal Pradesh, respectively. 3701 pigs died due to ASF (Table-4 and Figure-4) [11].

## Host Range and Transmission

ASFV infects members *Suidae* family such as domestic and wild boars, feral pigs, bush pigs, warthogs, forest hog, and *Ornithodoros* ticks. However, all wild pigs: Warthogs (*Phacochoerus aethiopicus*), bushpigs (*Potamochoerus porcus*), and ticks of *Ornithodoros* genus act as reservoir hosts, maintaining the virus thereby transmitting to the domestic pigs in Africa [33-35]. ASF is non-zoonotic disease. It may be hypothesized that contamination of the habitat by the infected and/ or dead wild pigs in Arunachal Pradesh and Assam or through movement of men and material from the infected habitat might be the possible route of transmission to domestic pigs in India. The role of ticks (*Ornithodoros* spp.) in transmission of the virus in pigs in India is yet to be established.

## Risk Factors

The most important risk factor associated with occurrence of disease in NE states of India is that many states share a porous border with neighboring countries such as Myanmar, Bangladesh, Tibet, and China wherein ASF is reported. There are no restrictions in the movement of men and material including piglets without any health records along the borders of Myanmar, Bangladesh, and Tibet. Other factors which may facilitate virus transmission such as location of pig farms, densely populated stys, seasons/marshy areas favoring breeding of ticks, improper disposal of carcass, slaughtering of pigs by farm side, sale of sick pigs, unknown source of replacement stock, feeding of swill, and unrestricted movement of men and materials between farms, vicinity of farm to forest areas wherein wild pigs come in contact with farm pigs [36,37]. Role of ticks in the transmission of ASF in India is yet to be reported.

## ASF Transmission in India [11]

A total of 11 outbreaks were reported in two states, namely, Arunachal Pradesh and Assam. The date of start of first outbreak was January 26, 2020, and was continuing till April 24, 2020. There were four outbreaks in Arunachal Pradesh and seven in Assam.

### Arunachal Pradesh

ASF outbreak reported in two districts, namely, East Siang and Papum Pare which are located 299 km (by road) apart. It is contemplated that the first outbreak of ASF was started in Pasighat town area on January 26, 2020, and then in Bilat area of East Siang district. The exact cause of origin of occurrence of disease could not be arrived as we could not make a visit to the site due to pandemic. It was learnt that local people could notice the death of wild boars in the drainages/rivulets nearby areas of Pasighat region that could have contaminated the habitat. Pasighat is located 25 km (by road) away from Bilat region. Simultaneously, disease was transmitted to Naharlagun region of Papum Pare district which is located 233 km away from Bilat region of East Siang. Approximately, after 3 months of appearance of disease in Naharlagun region, disease was transmitted to Nirjuli/Doimukh area of Papum Pare which is 8 km away from Naharlagun region. It may be hypothesized that movement of men and material between the regions along with transportation of piglets to these two regions might have caused the spread of disease.

### Assam

A total of seven outbreaks of ASF were reported in the state between February 24, 2020, and April 10, 2020. Since its first outbreak in Arunachal Pradesh region, after 1 month, the disease was reported in Dhemaji region (February 24, 2020) of Assam. Subsequently, after a week, disease was seen in Sivasagar region (March 7, 2020), then into Biswanath (March 20, 2020), again into another region of Sivasagar (March 23, 2020), and simultaneously (April 2, 2020) into different regions of Kamrup Metro and Sivasagar districts. On April 10, 2020, one more outbreak was seen in Jorhat districts of Assam. Sivasagar district of Assam reported three outbreaks in different regions within 26 days. It was noticed that the spread of disease was more rapid to different regions/districts which may be due to the panic among the farmers thereby opting for distress sale of pigs at less rates and also buying of pigs by other farmers/consumers at lower prices.

It may be hypothesized that within 3 months of period, all 11 outbreaks have happened as one cluster. Role of ticks in transmission of disease need to be established, otherwise fourth cycle (proposed), namely, wild boar-habitat cycle [22] might have played (hypothetical) role in outbreak of ASF in these two districts.

## Clinical Signs and Gross Pathology

There are different forms of clinical presentations of ASF, namely, peracute, acute, subacute, and chronic associated with virulence of virus Yoo *et al*. [38]. ASF virus isolates are classified as highly virulent, moderately virulent, and low virulent Pan and Hess [39]. The incubation period of the disease vary from 4 to 19 days. Mortality rate depends on virulence of virus ranging from 100% in disease caused by highly virulent to <20% in chronic forms caused by low virulent virus [26].

Peracute form of ASF caused by highly virulent strains of ASFV causes death within 4 days of infection without any gross lesions. Such form of disease is seen when virus enters into naïve pig farms. Infected pigs show high fever (up to 42°C), anorexia, lethargy, and sudden death and some pigs may show respiratory signs due to high temperature [40].

Acute form of ASF is caused by highly virulent virus and characterized by high fever (40-42°C), with mortality rate reaching up to 100% within 4-9 day’s post-infection [41]. The infected pigs show anorexia, lethargy, inactive, and bunch up together [42]. Bluish-purpled areas/hemorrhagic spots on ears, abdomen, hind legs, and generalized reddening of skin (chest, abdomen, tail, and leg), blood from nose/mouth, and bloody feces [26]. Lesions in dead pigs show pulmonary edema, hyperemic splenomegaly, extensive hemorrhages of internal organs, intensive necrosis of lymphoid tissue, petechial hemorrhages in the lungs, and urinary bladder and kidneys [43]. The other most important lesion described in acute ASF is multifocal hemorrhagic lymphadenitis. Lymph nodes can have multifocal or extensive hemorrhages that can produce a marbled appearance [42]. It may be contemplated after observing the mortality patterns in Assam and Arunachal Pradesh that acute form of the diseases was seen in pigs.

Subacute form of ASF is produced by moderately virulent strain and the clinical signs produced are milder ones which may overlap with other conditions in pigs and may not lead to suspicion of ASF. The clinical signs exhibited are similar to acute form of disease but less marked. Illness may last for 30-45 days. Most cases recover after intermittent fever up to 1 month. Mortality may vary from 30% to 70% and pigs may die after 20 days after infection [26,43]. The vascular changes observed in subacute forms of ASF are mainly hemorrhage and edema and are more intense than those reported in the acute form of the disease [44,45]. The pigs after recovery may excrete the virus up to 6 weeks [40]. There are two different stages that cause death of affected pigs: In the intense thrombocytopenia/leukopenia phase or in the recovery phase when hemorrhages appear due to erythrodiapedesis by vasodilation, especially in young animals [45,46].

Chronic form of ASF is produced by low virulent strains [40,42] with low mortality (20%) and no vascular lesions. This type of disease is seen in Spain, Portugal, and also in Dominican Republic. The chronic form may have arisen due to evolution of ASF virus isolates used in the vaccine trials carried out in Iberian Peninsula [40]. Various signs such as loss of weight, reddening of skin, growth retardation in growing pigs, irregular peaks of temperature, joint swellings, and respiratory signs. These viruses are non-haemadsorbing strains, but some animals develop discrete lesions in the lungs or on the skin over bony protrusions and joint swellings [26]. Recovered and infected pigs both by high and low virulent virus act as persistent carriers which transmit the disease in disease-free zones [20,41,43].

Diseases that are to be differentiated from ASF are Classical swine fever, Highly Pathogenic-Porcine Reproductive and Respiratory Syndrome, Aujeszky’s disease, Swine Erysipelas, Septicemic Salmonellosis, Porcine Dermatitis and Nephropathy Syndrome [40].

## Pathogenesis

**Figure-5:**
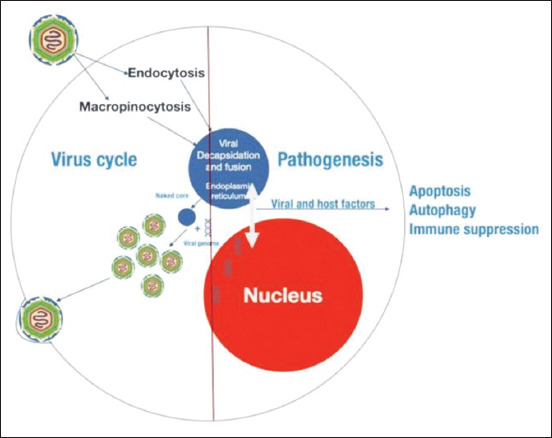
Replication cycle of African swine fever virus in a host’s cell.

ASF is a complex disease causes immunodeficiency in affected pigs [47] The virus replicates (Figure-5), after entering through oral-nasal route, initially in the tonsils or regional lymph nodes [48]. The cellular receptors and virus ligands still remains elusive [49]. Virus spreads to secondary organs of replication through blood (viremia) or lymph within 2-3 day’s post-infection [50], and later to other organs where it can replicate [45]. ASF virus targets mainly monocytes and macrophages [51], UK and NL genes of ASFV play a pivotal role in virulence and pathogenesis along with 8DR which is responsible for hemadsorption (HAD) property of HAD isolates which cause acute disease as compared to non-HAD [52]. The infected monocyte-macrophage appears swollen. There will be a necrosis in the infected cells and virions are released by budding, and can be observed free in the blood, lymph, and the interstitial tissue [53]. ASFV-induced apoptosis or necrosis causes the destruction of monocytes-macrophages. In acute form, lymphoid organs including spleen, lymph nodes, thymus, and tonsils are destroyed [51] and major population of B and T lymphocytes and macrophages undergo cell death [51]. Hemorrhagic or hyperemic splenomegaly, petechial and ecchymotic hemorrhages in multiple organs, pulmonary edema, and disseminated intravascular coagulopathy are the changes observed [26].

In subacute ASF, additionally, a more marked edema, ascites, and hydropericardium are observed [54]. Lymph nodes associated with kidney and gastrohepatic regions without fixed vascular macrophage population have severe hemorrhages [45]. Pulmonary intravascular macrophages get infected with virus causing hemorrhages and edema [53].

## Diagnosis

ASFV infection is very difficult to differentiate from CSFV infection either by clinical or post-mortem examination. Hence, it is essential to confirm the ASFV infection through laboratory diagnosis only. The laboratory diagnostic techniques are directed to detect the agent or immune response to agent. The samples to be sent for laboratory diagnosis are blood, serum, and tissues (spleen, lymph nodes, bone marrow, lung, tonsil, and kidney) [38]. The samples should preferably be transported on ice to laboratory.

### Detection of the agent

i. Virus isolation: Primary leukocyte culture or porcine bone marrow cells are commonly used for isolation of virus [26].
ii. HAD test [55]: Virus isolation in macrophages and HAD is the gold standard for identification of ASFV required for the first detection of ASF in disease-free regions or in the primary outbreak areas. 8DR protein of ASFV is responsible for HAD property wherein virus in macrophages binds to pig erythrocytes causing rosette [26] and some viruses are non-HAD. HAD ASFV are pathogenic and non-HAD ASFV are non-pathogenic or avirulent [38]. HAD test is cumbersome and is practiced in reference laboratories only. A detailed test protocol is available in OIE manual [43]. The HAD phenotypes of ASFV readily show CPE, where care should be taken while interpreting the non-HAD phenotypes of ASFV which also produce CPE but do not hemadsorb [56].
iii. Fluorescent antibody test (FAT): Directly can be used on field samples or those inoculated at the laboratory. Alone can serve as only presumptive diagnostic test along with clinical sings and typical lesions of ASFV [14].
iv. ELISA for antigen detection: The sensitivity of the assays is lower than polymerase chain reaction (PCR) or HAD. Hence, usually this method is not recommended for confirmatory diagnosis.
v. Conventional PCR: Should be performed using primer sets targeting the most conserved genome parts so as identify and detect a wide range of ASFV isolates of all the known genotypes [57].
vi. Real-time PCR: The technique is highly sensitive and less time-consuming and even can be used on samples which are not fit for virus isolation or antigen detection assays. The OIE recommends to follow quantitative PCR using real-time PCR primers and probes [34,58].

### Detection of immune response to agent or serological tests

The serological tests are the most commonly used diagnostic tests because of their simplicity, relatively low cost, and less specialized equipment’s are needed. The serological tests are also more relevant because as on date there are no commercially available vaccines and hence vaccination is not practiced. Which means presence of antibodies against ASFV indicates either disease is ongoing or it affected the herd. The antibodies appear as early as 7-10 days and can persist for several months or even years [26,56].

i. ELISA: The indirect ELISA [59] which is suitable for testing both serum and plasma. However, positive samples should be confirmed by indirect FAT (IFAT), indirect immunoperoxidase test (IPT) or immunoblotting. Different types of ELISA (competitive or blocking) commercially available are being used for the detection of antibodies against ASFV infection [56].
ii. IPT: The test developed by Gallardo *et al*. [60] is recommended by OIE.
iii. IFAT: The protocol developed by Sánchez-Vizcaíno [61] is recommended by OIE. IPT and IFAT are a confirmatory test for sera from areas that are free from ASF and are positive in the ELISA, and for sera from endemic areas that give an inconclusive result in the ELISA.

#### Immunoblotting test

The procedure standardized by Pastor [62] is used as alternate to IFAT and IPT to confirm equivocal results with individual sera. The tests perform better than other tests even in the detection of weak positive samples for ASFV antibodies.

## Prevention and Control

### Antiviral agents/compounds

Antiviral compounds/molecules can be of use in targeting virus-specific enzymes that play a role in viral replication including DNA polymerases of ASFV and some of the researchers have successfully used such drugs in preventing ASFV replication *in vitro* [63]. Sodium phenylbutyrate has shown inhibition of ASFV replication and viral late protein synthesis [64], Resveratrol and oxyresveratrol have shown an antiviral effect on ASFV in cell culture [65]. 5-(Perylen-3-ylethynyl)-arabino-uridine and 5-(Perylen-3-ylethynyl) uracil-1-acetic acid against ASFV have shown dose-dependent inhibitory effect on ASFV infection in Vero cells [66]. All these experiments were conducted *in vitro* and are to be validated in pigs.

### Biosafety and biosecurity measures

Biosafety at farm level is to be practiced. Persons/labors handling the infected pigs should take all biosafety precautions such as wearing of protective equipment such as aprons, spectacles, gloves, and gumboots and should not visit the other sheds. Gumboots should be washed with 2% sodium hydroxide immediately after use and various commercial products of disinfectants are available in the market [67].

Biosecurity at farm and village/surrounding regions is to be practiced. Isolation, restriction of movement and sanitation (cleaning and disinfection) has controlled the spread of ASFV [68]. Rapid culling of all infected and in-contact pigs and proper disposal of cadavers, litter, and waste food is essential. All these things should be buried deeply in the vicinity over layered with lime and salt, not to transport to distant places to avoid spillage. Thorough cleaning (with water) of farm/infected area and disinfection (Disinfection may be carried out with 2% sodium or calcium hypochlorite/sodium hydroxide or a detergent-based virucidal agent), [67] if tick population is high, one can use acaricide depending on the need. Farm utensils should be cleaned with detergents and washed properly. Creating awareness among the animal health workers about the disease, training them in early recognition, collection, and dispatch of suspected clinical samples, and intimation to the nearest dispensary are important steps in the field of health-care system [69].

## Vaccines

At present, there are no vaccines available commercially. Testing of infected and in contact pigs, ­culling all positive reactors are the only way of preventing the spread of infection, but that eliminates large number of pigs in the locality leading to economic losses to pig farmers. DNA vaccines that contain ASFV genome constructs devoid of CD2v, p54, and p30 have been tried and given limited protection in a population. The development of vaccines against ASF is difficult because of complex nature of virus with its large number of proteins evading host-immune system. Live-attenuated candidate vaccine strains have been generated from virulent strains and are under field trials (Table-7) [70]. Another obstacle in the ASFV propagation is non-availability of a permanent cell lines which can sustain its multiplicity and production in large scale [71].

**Table 7:** Details of attenuated vaccines against ASF under experimental stage [70].

S. No	Experimental Vaccines using different genotypes	Target gene (s)
1.	*+[72]	A238L
2.	*+[72]	A224L
3.	*+[72]	EP153R
4.	*+[73,74]	MGF360/530/505
5.	*+[75]	DP148R
6.	*$[76]	CD2v (EP402R)
7.	^[77]	9GL and UK
8.	^−[78]	TK
9.	^−[79]	9GL and MGFs
10.	+[80]	9GL
11.	^+[81]	MGF360/505
12.	^+[82]	9GL
13.	*+[83]	Naturally attenuated
14.	*+[84,85]	Naturally attenuated
15.	^#[86]	Low virulent
16.	*+[87]	Cell culture-adapted

* Genotype I;

^ Genotype II;

+ Protection against homologous challenge;

$ Protection against homologous and heterologous challenge; −No protection;

# Protection against virulent challenge

## Conclusion

ASF is a highly contagious disease of pigs and wild boars causing 100% mortality leading to huge economic loss to pig farmers. There is no vaccine available commercially. Affected and in contact pigs are to be culled and buried deep. The infected sheds and premises need to be cleaned and disinfected thoroughly and restocking can be done 4 months later from known source of healthy farms. The ASF is reported from neighboring countries of NE states of India and may enter at any time that can persistently remain in our pigs. Therefore, one need to be vigilant and alert.

While submitting this manuscript, OIE reported first occurrence of ASF in two states of India, namely, Assam and Arunachal Pradesh on May 21, 2020.

## Authors’ Contributions

SSP conceptualized, designed, collected all relevant literatures, and prepared the first draft. KPS collected ASF outbreak data and prepared the distribution map. VV prepared the topic relevant to epidemiological aspect of ASF. AP assisted in formatting the manuscript and arranging references as per the style. BP provided the information on studies related to ASF vaccines. PR edited the whole manuscript and provided the inputs required for finalizing the manuscript. All authors have reviewed the final manuscript and approved the same.
